# Submental Intubation Including Extubation: Airway Complications of Maxillomandibular Fixation

**DOI:** 10.1155/2012/841051

**Published:** 2012-12-12

**Authors:** Santosh Kumar Yadav, Gopendra Deo

**Affiliations:** ^1^Department of Oral and Maxillofacial Surgery, Chitwan Medical College, P.O. Box 42, Bharatpur-10, Chitwan, Nepal; ^2^Department of Anesthesiology, Chitwan Medical College, P.O. Box 42, Bharatpur-10, Chitwan, Nepal

## Abstract

Hernandez first described the submental route for endotracheal intubation in 1986 as an alternative airway maneuver for maxillofacial procedures. Since that time, several case studies have been performed demonstrating the efficacy of the submental approach. This method was recently implemented in the case of a patient with altered nasal anatomy who sustained a mandibular fracture necessitating maxillomandibular fixation. Unlike most of the cases described in the literature, this patient's operative course was confounded by the need to extubate through the submental tunnel. The patient tolerated the procedure well and was able to avoid other forms of surgical airway.

## 1. Introduction 

Several procedures exist in which the surgeon requires access to areas that would otherwise be obscured by an endotracheal tube. Means of securing a patient's airway other than the conventional oral intubation include nasotracheal intubation and surgical airways such as tracheostomy. In 1986, Hernandez described submental intubation as an alternative to the classic methods [1]. His technique consisted of passing the endotracheal tube through the anterior floor of the mouth to allow free intraoperative access to the dental occlusion and to the nasal pyramid without endangering patients with skull base trauma [2]. This technique allows for the avoidance of tracheal dissection and eliminates the risks associated with nasotracheal intubation in the setting of facial trauma. 

As described in a paper by Caubi et al., the surgeon performs the procedure by creating a small incision just medial to the lower border of the mandible. The subcutaneous tissues and the tissues of the floor of the mouth are bluntly dissected through with Kelly forceps to the point of entering the oral cavity just anterior to the sublingual caruncle. Care is taken to avoid nerves, vessels, and salivary structures. The endotracheal tube is then disconnected from the ventilatory circuit and is pulled through the submental tunnel, along with the pilot balloon, using the forceps [3]. The circuit is then quickly reconnected and tube positioning is confirmed by comparing the position of the tube with regard to the teeth before and after submental positioning as well as confirming bilateral breath sounds. At the end of the operation, the tube is disconnected, pulled back into the oral cavity and reconnected. The submental incision is sutured and the patient is extubated in the usual fashion [2, 4]. 

Several case studies have been performed since the introduction of the procedure in 1986. These reports consistently show no motor or sensory deficit, normal healing of the mucosal floor, and preservation of the salivary ducts and saliva production [2, 5]. Of the complications experienced, one report by Meyer et al. found an 8% occurrence of oral floor abscesses and a 4% occurrence of hypertrophic scar formation. However, the patient population in this series was rather small (*n* = 25) [2]. Caubi et al. describe an intraoperative complication in only one of their study patients in which high pressures where observed due to compression of the endotracheal tube as a result of the acute angle the tube takes in the oropharynx [3]. Despite long-term airway support and maintenance being listed as a contraindication to submental intubation in the MacInnis and Baig trial, they did observe one patient who remained intubated in the ICU for 3 days and found that delayed extubation had been uncomplicated in all cases where it was required [6].

The consensus among the various reports all concludes that the complications are minimal and that patients are overall very satisfied with the lack of scarring. In addition to the obvious benefits of submental endotracheal intubation in maxillofacial trauma procedures, elective use of this maneuver has been described as efficacious in procedures where an unobstructed oral or nasal cavity is beneficial to the surgeons, such as orthognathic surgery and even transfacial cranial base surgery [4, 7]. 

## 2. Case Report 

The patient was a 30-year-old, 60 kg male who presented several days after sustaining blunt trauma to the left side of his jaw. The patient was playing football and was elbowed in this area. He immediately had swelling and pain in the left mandibular area adjacent to his ear. He had limited jaw mobility, which greatly exacerbated his pain and was only able to tolerate eating soft foods. On admission, the patient had obvious facial swelling to the left side of his face and dental misalignment was noted. A maxillofacial CT scan was obtained which showed an acute left mandibular fracture just inferior to the condyles. The remaining facial bones were intact without acute fracture. Also observed on the CT scan were narrow nasal passages and a leftward deviated nasal septum. Otolaryngology scheduled surgical intervention for fracture reduction and maxillomandibular fixation. 

Evaluation preoperatively showed poor mouth opening secondary to pain. He was appointed a Mallampati score of II and was estimated to have a thyromental distance greater than 6 cm. Nasotracheal intubation was the proposed means of airway control due to the necessity of maxillomandibular fixation thus precluding orotracheal intubation. The patient demonstrated patency of both nares and provided no history of nasal trauma or knowledge of any nasal septal deviation. The patient was premedicated with midazolam 2 mg intravenously and fentanyl 100 mcg intravenously. All standard monitors were applied. The patient received 3 sprays of oxymetazoline to each naris. Anesthesia was induced with propofol 200 mg intravenously and fentanyl 150 mcg intravenously. After successful mask ventilation, succinylcholine 100 mg was administered intravenously. Easy mask ventilation was achieved with confirmation of end-tidal CO_2_ and adequate oxygenation. A nasal RAE endotracheal tube was then introduced into the right naris with immediate resistance. Repositioning of the angle of entry did not overcome the difficult passage, nor did downsizing to a smaller nasal RAE endotracheal tube. Intubation via the right naris was aborted and intubation through the left naris was attempted. However, immediate resistance was again experienced and it was felt at this time that nasotracheal intubation was not going to be possible. The patient was mask ventilated again and was then intubated orally with an 8.0 endotracheal tube. On direct laryngoscopy, miniscule amounts of blood were noted in the patient's pharynx from the nasal trauma sustained during the initial intubation attempts. Bilateral breath sounds and end-tidal CO_2_ were confirmed. The surgeon then made a small, approximately 2 cm incision in the right submental region and bluntly dissected through the floor of the mouth with Kelly forceps. The ventilator was then briefly disconnected and the endotracheal tube and pilot balloon were pulled through the submental tunnel via the Kelly forceps. The circuit was reconnected, end-tidal CO_2_ and bilateral breath sounds were confirmed, and direct laryngoscopy was repeated to confirm tube placement. The supraglottic and oropharyngeal areas were adequately suctioned and the surgeons proceeded with the case. Anesthesia was successfully maintained with inhaled isoflurane. The patient's initial airway pressures while orotracheally intubated were consistently 15-16 cm H_2_O. However, after tunneling the endotracheal tube through the floor of the mouth, peak pressures increased to 25–30 cm H_2_O. These increased pressures were felt to be secondary to the acute bend that the tube was required to make in the oropharynx. No other issues with ventilation or oxygenation were encountered throughout the case. The surgical team inserted four, 8 mm self-drilling screws into the patient's maxilla and mandible and used 26-gauge wire to achieve properly aligned dental occlusion. The submental incision was then infiltrated with lidocaine and epinephrine. A soft suction catheter was used to attempt to remove as much secretions as possible from the patient's pharynx. The inhalational agent was turned off and the patient awakened. The surgical team was standing by with wire cutters in hand should airway compromise had been encountered. It was felt that the patient was adequately awake when he was able to generate sufficient and consistent tidal volumes, follow commands to squeeze fingers and maintain a head lift when asked. At this time the cuff was deflated and the endotracheal tube was slowly removed through the submental tunnel. The surgical team closed the submental incision, first with 4-0 Vicryl for the subcutaneous plane and a 5-0 fast absorbable suture for the skin. The patient maintained excellent ventilation and oxygenation and at no time experienced any airway compromise or oxygen desaturation. He was taken to the postanesthesia care unit in excellent condition. 

## 3. Discussion 

Submental intubation has been heralded as a simple, secure, and effective procedure for operative airway control in major maxillofacial traumas. It allows practitioners to avoid the risk of epistaxis, iatrogenic meningitis, or trauma of the anterior skull base after nasotracheal intubation, as well as complications, such as tracheal stenosis, injury to cervical vessels or the thyroid gland, subcutaneous emphysema, or recurrent laryngeal nerve injury related to tracheostomy [3, 8]. The scar from the submental incision is thought to be less visible than a tracheostomy scar and has been well tolerated by patients [2]. In addition to possible hypertrophy of the submental scar, other complications include accidental extubation, accidental advancement of the endotracheal tube into a mainstem bronchus, bleeding, infection, mucocele formation, and damage to salivary ducts and glands. However, these complications have proven to be quite rare according to data from various trials over the past 24 years. In our particular case, the submental intubation approach allowed for appropriate alignment and maxillomandibular fixation, all while maintaining a secure airway. We did experience an increase in peak airway pressures, which were attributed to the compression of the endotracheal tube as it acutely bends in its path through the oropharynx and into the larynx. However, the increase in pressure was not significant enough to warrant us attempting to exchange the endotracheal tube for a reinforced endotracheal tube with the included risk of possibly losing control of the airway. In addition, unlike the great majority of cases reported in the literature, we were not afforded the luxury of being able to reverse the submental intubation into a regular oral intubation at the end of the case due to the surgical fixation. Therefore, absolute assurance of the patient's ability to protect his airway was necessary prior to submental extubation. Any complications occurring after submental extubation, such as emesis with aspiration or laryngospasm, would have proven disastrous, as the patient had undergone fixation of his mandible and was unable to open his mouth. The importance of communication and cooperation between the anesthesia and surgical teams cannot be overemphasized in this situation, as the surgical team was ready to immediately clip the fixation wires to allow for jaw opening, suction, and emergent reintubation if needed. Only after the patient had as much of his oropharyngeal secretions suctioned as possible and was completely and purposefully following commands, he was extubated with immediate suturing of his submental incision. 

Alternative routes of management for this patient included not performing the procedure at all, repeated attempts at nasotracheal intubation with a smaller endotracheal tube and fiber optic laryngoscopy, or tracheostomy. Not performing the procedure would have caused the patient great morbidity in terms of pain, trismus, possible malunion, and permanent defects in mastication secondary to poor healing. Therefore it was felt that the procedure must be performed. Repeated attempts at nasotracheal intubation could have been performed, but not without the risks of further nasal mucosal trauma and increased bleeding. Again, as the patient was to have maxillomandibular fixation, any increased amount of blood in the glottic area would put the patient at higher risk for laryngospasm, and thus, no further attempts were felt to be in the patient's best interest. Tracheostomy is another form of a surgical airway, but carries the risk of possible tracheal stenosis and damage to surrounding tissues, vessels, and nerves. Also, the scar of tracheostomy is more visible than that of the submental incision, which is hidden on the underside of the mandible. After all of the above alternatives had their risks and benefits weighed, we felt that submental intubation was properly indicated for this particular case. 

Our patient, like those described in the existing literature, tolerated the procedure well and had completely successful management of his airway, as well as uncomplicated extubation, with the submental endotracheal airway management approach. 

What lacks in the current literature are any large-scale case series or prospective trials comparing the efficacy of submental intubation to that of other types of surgical airway. This type or airway management is even being used electively for certain types of procedures including skull base surgery. It is difficult to truly assess the overall incidence of complications that occur with submental intubation when most case series are only reporting on 8–10 cases. The current data must be interpreted with caution and much more research into this area of airway management is warranted. 
